# Identifying the risk factors for intracranial herniation in patients with cerebral venous thrombosis

**DOI:** 10.1055/s-0043-1767822

**Published:** 2023-05-31

**Authors:** Yasemin Dinç, Rıfat Ozpar, Bahattin Hakyemez, Mustafa Bakar

**Affiliations:** 1 Uludağ University, Faculty of Medicine, Department of Neurology, Bursa, Türkiye.; 2 Uludağ University, Faculty of Medicine, Department of Radiology, Bursa, Türkiye.

**Keywords:** Sinus Thrombosis, Intracranial, Brain Edema, Intracranial Hypertension, Trombose dos Seios Intracranianos, Edema Encefálico, Hipertensão Intracraniana

## Abstract

**Background** Cerebral venous sinus thrombosis (CVST) is not as well understood as an ischemic stroke of arterial origin. Although the prognosis of CVST is usually good, parenchymal lesions may occur in some patients, and the development of intracranial herniation may result in death. For this reason, recognizing the risk factors for intracranial herniation and accurately determining those patients who should undergo decompressive craniectomy is important.

**Objective** This study aims to determine the risk factors for intracranial herniation in patients with CVST.

**Methods** A total of 177 patients diagnosed with CVST between 2015 and 2021 in our tertiary center were retrospectively included in this study.

**Results** Of the 177 patients, 124 were female and 53 were male with mean ages of 40.65 ± 13.23 and 44.13 ± 17.09, respectively. Among those, 18 patients had developed intracranial herniation. A significant statistical relationship was observed between superior sagittal sinus thrombosis, sinus rectus thrombosis, venous collateral score, nonhemorrhagic venous infarct, presence of malignancy, small juxtacortical hemorrhage, and cortical vein thrombosis. The binary logistic regression analysis results showed that the most significant variables were the venous collateral score of 0, malignancy, and small juxtacortical hemorrhages.

**Conclusion** This study identified small juxtacortical hemorrhages, the presence of malignancy, and a venous collateral score of 0 to be independent risk factors for intracranial herniation in CVST patients. Drawing on these results, we recommend close clinical observation of CVST patients, as they may be candidates for decompressive craniectomy.

## Resumo

**Antecedentes** A trombose do seio venoso cerebral (CVST) não é tão bem compreendida como um acidente vascular cerebral isquémico de origem arterial. Embora o prognóstico de CVST seja geralmente bom lesões parenquimatosas podem ocorrer em alguns pacientes e o desenvolvimento de herniação intracraniana pode resultar em morte. Por esse motivo é importante reconhecer os fatores de risco para hérnia intracraniana e determinar com precisão os pacientes que devem ser submetidos à craniectomia descompressiva.

**Objetivo** Este estudo tem como objetivo determinar os fatores de risco para herniação intracraniana em pacientes com CVST.

**Métodos** Um total de 177 pacientes diagnosticados com CVST entre 2015 e 2021 em nosso centro terciário foram retrospectivamente incluídos neste estudo.

**Resultados** Dos 177 pacientes 124 eram do sexo feminino e 53 do masculino com média de idade de 40 65 ± 13 23 e 44 13 ± 17 09 respectivamente. Destes 18 pacientes desenvolveram hérnia intracraniana. Uma relação estatística significativa foi observada entre trombose do seio sagital superior trombose do seio reto escore de colateral venosa infarto venoso não hemorrágico presença de malignidade pequena hemorragia justacortical e trombose da veia cortical. Os resultados da análise de regressão logística binária mostraram que as variáveis mais significativas foram o escore colateral venoso de 0 malignidade e pequenas hemorragias justacorticais.

**Conclusão** Este estudo identificou pequenas hemorragias justacorticais a presença de malignidade e um escore colateral venoso de 0 como fatores de risco independentes para herniação intracraniana em pacientes CVST. Com base nesses resultados recomendamos uma observação clínica rigorosa dos pacientes CVST pois eles podem ser candidatos à craniectomia descompressiva.

## INTRODUCTION

Cerebral venous sinus thrombosis (CVST) is an uncommon type of stroke caused by the blockage of the dural venous sinuses and cortical veins. 1 2 3 4 This condition has been identified in less than 0.5% of ischemic stroke cases, and its annual incidence has been reported to be 4 to 6 per million people. 5 6

In recent years, CVST has been exhibiting good clinical outcomes as a result of early diagnosis and treatment. However, despite early administration of anticoagulation treatment, neurological deterioration and parenchymal lesions may occur in some patients. These conditions may be associated with poor clinical outcomes and mortality. 7 Moreover, supratentorial hemorrhagic venous infarctions and high intracranial pressure due to brain edema lead to brain herniation and neurological deterioration, which occurs in approximately 4% of patients. 8 Brain herniation is one of the most common causes of mortality in CVST patients. The term “malignant CVST” has been widely used to define this condition. 9

Although anticoagulation is considered the gold standard for recanalization by preventing thrombosis progression, it is an inadequate treatment for the current mass effect and high intracranial pressure in patients with malignant CVST. 9 Furthermore, since CVST may show clinical heterogeneity as well as ethnic and racial differences, identifying some risk factors may be useful in taking adequate precautions. Therefore, this study aims to determine the risk factors for intracranial herniation in CVST patients.

## METHODS

A total of 177 patients diagnosed with CVST in our tertiary center between 2015 and 2021 were retrospectively included in this study. Approval for this study was sought from the local ethics committee through a letter dated 06.07.2022, and it was granted (No. 2022-14/8). Patients' consent was not required given the retrospective nature of this study.

The inclusion criteria were diagnosis of CVST with contrast-enhanced cranial magnetic resonance imaging (MRI) venography, determination of the disease's etiology, and completion of monthly neurology outpatient controls for 3 months after CVST diagnosis. The exclusion criteria were withdrawal from neurology outpatient follow-ups and inability to undergo noncontrast cranial computed tomography (CT), cranial MRI, and contrast-enhanced MRI venography.

All medical records were stored in our medical operating system. As per the hospital's protocol, cranial MRI venography and cranial MRI were performed simultaneously. The cranial MRI venography was evaluated blindly by a neuroradiologist with 20 years of experience in the field, whereas the venous collateral scale was evaluated using contrast-enhanced cranial MR venography.

All images were evaluated for the presence of parenchymal lesions associated with the blockage of the dural sinus and cortical veins, which can be observed both in the venous collateral score (VCS) from the MRI venography and in the T2W sequences from the noncontrast CT and/or MRI. A VCS of 0 represents the lack of venous drainage in the brain parenchyma affected by CVST; a VCS of 1 represents the presence of a vein draining the affected area but not connected to an open sinus; and a VCS of 2 represents the connection of a vein draining the area affected by CVST to an open sinus. 10 Parenchymal lesions were classified into hemorrhagic and nonhemorrhagic venous infarcts. Furthermore, hemorrhagic venous infarcts were evaluated in terms of three categories—subarachnoid hemorrhages, small juxtacortical hemorrhages, and large parenchyma hematomas. 11 The patients' complaints, while being examined by a neurologist in the emergency room, were classified into three categories, namely, isolated intracranial hypertension, focal neurological deficits, and epileptic seizures, and these complaints were analyzed. Next, the patients were admitted in the neurology department where anticoagulant medication was initiated. In the case of neurological deterioration, the patients underwent control-cranial CT under emergency conditions.

The patients were also evaluated for rheumatologic diseases and hypercoagulopathy. After being discharged from the hospital, their neurological examination results and modified Rankl scores (mRs) were calculated and recorded in the epicrisis. The radiological features of intracranial herniation were defined as uncal herniation and as a midline deviation of ≥ 5 mm. Decompressive craniectomy was performed in patients with intracranial herniation with Glasgow coma scale scores of < 8. The clinical outcomes of the patients were evaluated during the third month in the neurology outpatient clinic using the mRs (mRs 0–2 indicates good clinical outcome; mRs 3–6 indicates poor clinical outcome).

Moreover, patients with and without intracranial herniation were compared in terms of their clinical demographics and radiological features.

### Statistical analysis

The clinical, demographic, and radiological information of the CVST patients with or without intracranial herniation were compared. Statistical analysis was conducted using the Statistical Package Social Sciences (SPSS, IBM Corp., Armonk, New York, USA) version 23.0, and the MedCalc statistical software (MedCalc LTD., Ostend, Belgium0) version 19.1.5. A Shapiro–Wilk test was conducted to determine whether the data are normally distributed. The continuous variables are presented as means, standard deviations, and medians (25–75% quartiles), whereas the categorical variables are presented as frequencies and percentages. A two-sided Mann–Whitney U test or a two-sided independent sample t-test was applied to determine the difference or similarity between groups of continuous variables. Furthermore, the two-sided Fisher exact test and the Pearson chi-squared test were applied to the categorical variables to compare the differences between groups. Binary logistic regression was also performed, and the crude odds ratios (ORs), along with their 95% confidence intervals (CIs), were reported. A *p* -value < 0.05 was considered significant.

## RESULTS

This study involved 177 patients, of whom 124 (70.1%) were female and 53 (29.9%) were male, with mean ages of 40.65 ± 13.23 and 44.13 ± 17.09, respectively. Their mean ages were statistically similar ( *p* = 0.192). On the basis of clinical presentations of CVST, 122 (68.9%) patients were determined as having isolated intracranial hypertension, 27 (15.2%) were suffering from focal neurological deficits, and 28 (15.9%) were determined as having epileptic seizures. On the basis of etiology, CVST developed due to oral contraceptive use in 14 (7.9%) patients, pregnancy and puerperium in 34 (19.2%), rheumatologic disease in 26 (14.6%), prothrombic disease in 24 (13.5%), malignancy in 27 (15.2%), unknown etiologies in 28 (15.8%), head trauma and mechanical effects in 6 (3.3%), infection in 10 (5.6%), and other causes in 8 (4.5%) patients.

In terms of radiological features, 70 (39.5%) patients had superior sagittal sinus thrombosis, 129 (72.9%) had transverse sinus thrombosis, 104 (58.8%) had sigmoid sinus involvement, 8 (5%) had sinus rectus thrombosis, and 54 (30.5%) had jugular vein thrombosis.

**Figure 1:**
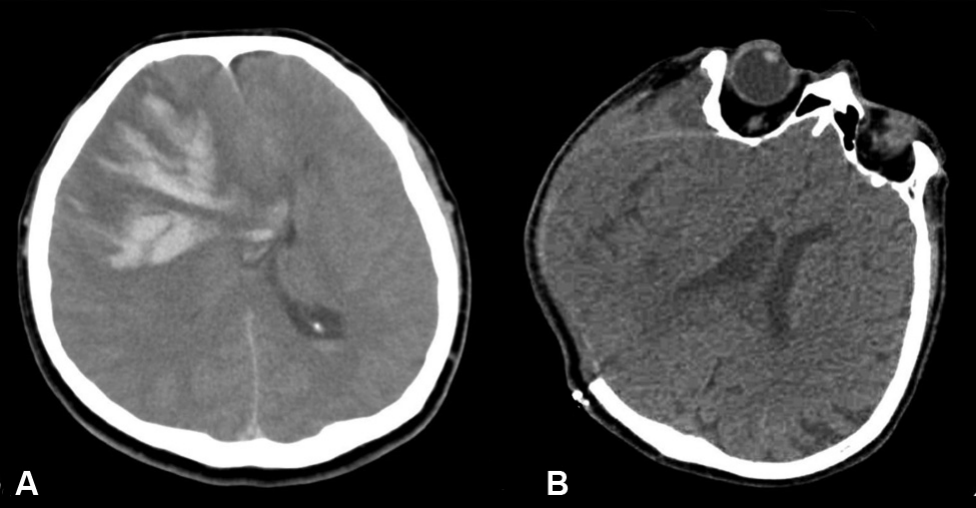
Example of a case with herniation due to intraparenchymal hematoma. ( **A** ) Reference cranial CT; 5.5 cm diameter parenchymal hematoma and herniation in the right frontal ventricle. ( **B** ) Cranial CT, right frontoparietal craniectomy view after decompression surgery.

**Figure 2:**
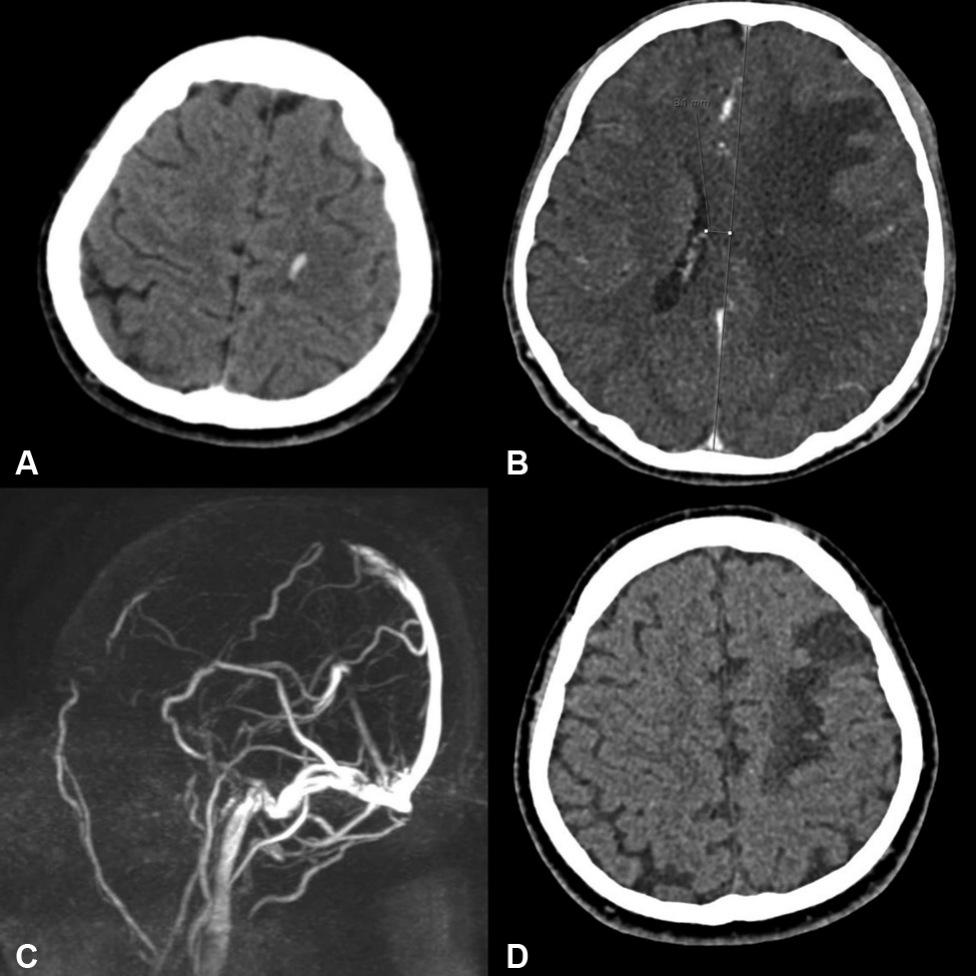
Example of herniated case with juxtacortical hemorrhage. On uncontrasted cranial CT, ( **A** ) cross-section through convexity left frontoparietal parasagittal juxtacortical hemorrhage, ( **B** ) section passing through the ventricular level subfalcian herniation from left to right. ( **C** ) Filling defect consistent with thrombosis and poor collateralization in the middle and anterior part of the superior sagittal sinus on MR venography. ( **D** ) The 3-month follow-up cranial CT showing hypodensity of venous infarction in the left frontal.

Examinations of the parenchymal lesions showed that 52 (29.3%) of the patients had nonhemorrhagic venous infarction, 12 (6.7%) had large parenchymal hematoma, 5 (2.8%) had subarachnoid hemorrhage, and 22 (12.4%) had small juxtacortical hemorrhages. Furthermore, isolated cortical vein thrombosis was present in 9 (50.8%) patients. A total of 42 (23.7%) patients had cortical vein thrombosis, in addition to dural sinus involvement. Overall, 51 (28.8%) patients were afflicted with cortical vein thrombosis. In terms of venous collateral scale, 37 (20.9%) patients had a score of 0, whereas 33 (18.6%) and 107 (60.4%) patients had a score of 33 and 2, respectively. In addition, 18 (10.1%) patients developed intracranial herniation, 15 of whom underwent decompressive craniectomy; 9 (5.1%) died due to CVST. There were 36 (20.3%) patients who exhibited poor clinical outcomes, whereas 141 (79.6%) or them displayed good clinical outcomes. Cranial imaging of two patients with intracranial herniation is shown in Figures 1 and 2 . When the demographic, etiological, and radiological variables associated with intracranial herniation were evaluated, a significant statistical relationship was observed between superior sagittal sinus thrombosis ( *p* = 0.013), sinus rectus thrombosis ( *p* = 0.019), venous collateral score ( *p* < 0.001), nonhemorrhagic venous infarct ( *p* = 0.004), presence of malignancy ( *p* < 0.001), small juxtacortical hemorrhage ( *p* < 0.001), and cortical vein thrombosis ( *p* < 0.001). Meanwhile, no significant statistical relationship was found between CVST etiology, transverse sinus thrombosis, inferior sagittal sinus thrombosis, sigmoid sinus thrombosis, jugular vein thrombosis, isolated cortical venous thrombosis, subarachnoid hemorrhages, and large parenchymal hematoma ( Table 1 ).

**Table 1:** Evaluation of demographic, radiologic, and clinical properties of cerebral venous sinus thrombosis patients with intracranial herniation or not

Variables		Patients with intracrainal herniation (n = 18)	Patients without intracrainal herniation (n = 159)	*p* -value
Age* (mean ± SD)		43.77 ± 2.22	41.45 ± 2,07	ns
Sex ** (female)		15(83.33%)	109(68,51%)	ns
**Clinical presentation**	Isolated intracranial hypertension**	6(33.33%)	116(72.95%)	**0.001**
	Focal neurological deficits**	9(50%)	18(11.32%)	
	Seizure at onset**	3(16.66%)	25(15.72%)	
**Risk Factors**	Pregnancy**	0(0%)	5(3.14%)	ns
	Postpartum**	2(11.11%)	19(11.94%)	ns
	Usage of oral contraceptive drug**	2(11.11%)	12(7.54%)	ns
	Prothrombotic disease**	5(27.77%)	48(30.18%)	ns
	Rheumatologic disease**	2(11.11%)	23(14.46%)	ns
	Behcet disease**	1(5.55%)	15(9.43%)	ns
	Malignancy**	6(33.33%)	17(10.69%)	**<0.001**
**Radiologic properties**	Superior sagittal sinus thrombosis**	12(66.66%)	58(36.47%)	**0.013**
	Transverse sinus thrombosis**	11(61.11%)	118(74.21%)	ns
	Inferior sagittal sinus thrombosis**	2(11.11%)	9(5.66%)	ns
	Sinus rectus thrombosis**	3(16.66%)	6(3.77%)	**0.019**
	Sigmoid sinuses thrombosis**	9(50%)	95(59.74%)	ns
	Jugular vein thrombosis**	7(38.88%)	47(29.55%)	ns
	Isolated cortical vein thrombosis**	2(11.11%)	7(4.40%)	ns
	Involvement of cortical vein thrombosis**	12(66.66)	39(24.52)	**<0.001**
Venous collateral scale**	VCS 0	12(66.66%)	25(15.72%)	**<0.001**
	VCS 1	2(11.11%)	31(19.49%)	
	VCS 2	4(22.22%)	103(64.77%)	
Parenchymal lesions**		13(%72.11)	49(30.81%)	**<0.001**
	Nonhemorrhagic venous infarction**	11(61.11%)	41(25.78%)	**0.004**
	Juxtacortical hemorrhages**	9(50%)	13(8.17%)	**<0.001**
	Subarachnoid hemorrhages**	0(0%)	5(3.14%)	ns
	Parenchymal hemorrhages**	2(11.11%)	10(6.28%)	ns

**Abbreviations:** ns, nonsignificant; SD, standard deviation; VCS, venous collateral score. **Notes:** Significant variables are shown in bold; *Mann-Witney U test; **Pearson chi-square test/continuity correction test/Fisher exact test.

When the significant variables associated with the development of intracranial herniation in CVST patients were analyzed using binary logistic regression, the most significant variables were found to be venous collateral score ( *p* = 0.006, OR = 6.078), presence of malignancy ( *p* = 0.021, OR = 5,490), and small juxtacortical hemorrhages ( *p* = 0.045, OR = 5.310) ( Table 2 ).

**Table 2:** Significant variables of intracranial herniation of patients with cerebral venous sinus thrombosis using binary logistic regression analysis

Variables	*p* -value	OR	95% CI	
			Lower	Upper
Superior sagittal sinus thrombosis (absent vs. present)	0.220	2.315	0.605	8.854
Sinus rectus thrombosis (absent vs. present)	0.127	4.198	0.665	26.498
Small juxtacortical hemorrhages (absent vs. present)	**0.045**	5.310	1.036	27.220
Nonhemorrhagic venous infarction (absent vs. present)	0.812	1.185	0.291	4.826
İnvolvement of cortical vein thrombosis (absent vs. present)	0.893	1.125	0.204	6.205
Venous collateral scores (VCS = 0 vs. VCS = 1–2)	**0.006**	6.370	1.701	23.865
Clinical presentation (isolated intracranial vs. focal neurological deficits and seizures)	0.110	3.078	0.774	12.243
Present of malignancy as a risk factor (absent vs. present)	**0.021**	5.490	1.299	23.197

**Abbreviations:** CI, confidence interval; OR, odds ratio; VCS, venous collateral score; vs, versus. **Notes:** Significance of the model: *p* < 0.001; significant variables are shown in bold.

## DISCUSSION

As an ischemic stroke of arterial origin, CVST is not as well understood due to its clinical heterogeneity and low incidence. Moreover, it is often difficult to diagnose and treat, and its clinical outcomes are difficult to predict. Although the prognosis of CVST is usually good, parenchymal lesions may occur in some patients and the development of intracranial herniation may result in death. 7 For patients with clinically worsening CVST, mechanical thrombectomy is advised. 12 However, a recent double-blind randomized study has shown that the efficacy of mechanical thrombectomy in CVST patients could not be demonstrated. 13

For this reason, it is important to identify the risk factors of intracranial herniation and determine those patients who should undergo decompressive craniectomy. The radiological features required for considering decompressive craniectomy are a large uncal herniation, a midline shift of ≥5 mm, and hypodensity due to herniation in the posterior cerebral artery region. Decompressive craniectomy should be performed only when aggressive medical management fails and when the time is inadequate for anticoagulation treatment to provide recanalization. 14

In this study, we found that the variables associated with intracranial herniation in CVST patients were superior sagittal sinus thrombosis, sinus rectus thrombosis, venous collateral score, presence of malignancy, and parenchymal lesions. However, our binary logistic regression analyses found that the venous collateral score, presence of malignancy, and small juxtacortical hemorrhages are the primary independent risk factors contributing to the development of intracranial herniation in CVST patients.

When transverse and sigmoid sinus thromboses occur, the presence of contralateral sinus can compensate for venous drainage, unlike in single midline thromboses such as superior sagittal sinus and sinus rectus. In addition, the superior sagittal sinus is the main sinus responsible for draining an empty space. Therefore, an increase in intracranial pressure is expected in superior sagittal sinus thrombosis. Small juxtacortical hemorrhages have recently been defined as concave hemorrhages measuring < 20 mm, with a high specificity but low sensitivity to CVST. 15 A later study has found that small juxtacortical hemorrhages are associated with superior sagittal sinus thrombosis and cortical vein thrombosis. 16 Cortical veins drain the brain parenchyma and carry the blood to the venous sinuses. Consequently, in their occlusion, the rise in intravascular hydrostatic pressure ruptures the intracortical region, causing small juxtacortical hemorrhages. 17 18 19

The relationship between intracranial hemorrhage and intracranial herniation is well established. 14 20 To our knowledge, this study is the first to determine the relationship between small juxtacortical hemorrhage and intracranial herniation. The reason behind this relationship could be the disruption of the blood–brain barrier and the rapid increase in vasogenic edema after the occurrence of small juxtacortical hemorrhage. 16 21 22 Another variable that was determined as an independent risk factor for intracranial herniation in this study was the venous collateral score. Since the brain parenchyma's drainage is impaired in the most distal part of a cortical vein thrombosis, a parenchymal lesion is likely to occur. However, in dural sinus thrombosis, the condition of the venous collaterals determines the clinical outcome. If the venous collaterals are sufficient, no parenchymal lesion will occur given that adequate drainage is possible. However, if collaterals are insufficient, parenchymal lesions may occur due to increased hydrostatic pressure, leading to herniation. 23 24 25 26

Another independent risk factor for intracranial herniation in patients with CVST identified in the current study was the presence of malignancy. The relationship between malignancy and poor clinical outcomes in patients with CVST has already been reported. 27 28 The presence of malignancy may cause CVST through several mechanisms—hypercoagulopathic states caused by tumors and chemotherapy drugs, tumor invasion into the dural sinus, and severe dehydration resulting from impaired oral intake and vomiting. A recent large case–control study has found that a history of cancer increases the risk of CVST, especially in patients with acute lymphocytic leukemia (ALL). The risk of thrombosis is the highest among other cancers. 27 The reason behind the high risk of intracranial herniation in patients with malignant CVST is the high clot load generated as a result of the prothrombotic state triggered by many mechanisms, such as late initiation of anticoagulant therapy in patients and damage to the blood–brain barrier. 29 30 Our results are consistent with those reported in the literature, 7 wherein poor clinical outcomes and death were more frequent in CVST patients with intracranial herniation.

### Limitations of the study

All patients included in this study had to undergo a 3-tesla MR venography. Thus, one of the limitations of this study is the exclusion of patients who could not undergo MRI. Therefore, the retrospective nature of this study is its most significant limitation.

In conclusion, this study identified independent risk factors for intracranial herniation in CVST patients, namely, small juxtacortical hemorrhages, presence of malignancy, and low venous collateral score. Drawing on these results, we recommend close clinical observation of CVST patients, as they can be candidates for decompressive craniectomy. Further multicenter and prospective studies must be conducted to obtain clearer information on the investigated condition.
